# Combining Small‐Molecular Compounds With CAR T‐Cell Therapy: Novel Strategies for Enhanced Cancer Immunotherapy

**DOI:** 10.1002/cai2.70053

**Published:** 2026-03-28

**Authors:** Rangzi Yi, Zijian Zhang, Yang Yang, Haichuan Zhu

**Affiliations:** ^1^ Department of Endocrinology, Tianyou Hospital, School of Medicine Wuhan University of Science and Technology Wuhan Hubei China; ^2^ National “111” Center for Cellular Regulation and Molecular Pharmaceutics, Key Laboratory of Fermentation Engineering (Ministry of Education) Hubei University of Technology Wuhan Hubei China

**Keywords:** CAR T, combined therapy, immunotherapy, molecular compound, T‐cell exhaustion

## Abstract

Chimeric antigen receptor (CAR) T‐cell therapy has been proved to be an effective cancer immunotherapy strategy against haematological malignancies, but exhaustion, limited persistence, and treatment‐related toxicity have been identified as major roadblocks in solid tumour treatment. Small‐molecular compounds could effectively improve CAR T‐cell therapy, such as preventing exhaustion, enhancing memory formation, and enhancing the antitumor activity. Additionally, adding small molecule switches based on tetracycline‐controlled gene expression system is an effective strategy to improve the safety of CAR T therapy and reduce its side effects. Despite the encouraging preclinical and clinical results, challenges still remain in optimizing dosing regimens and managing drug interactions. This review aims to summarize recent advances in combined approach and to discuss the underlying mechanisms of its potential benefits.

## Graphical abstract

Chimeric antigen receptor (CAR) T‐cell therapy, although revolutionary for haematological malignancies, faces significant challenges, including T‐cell exhaustion, limited persistence, and treatment‐related toxicity. The integration of small‐molecule compounds offers a promising strategy to overcome these limitations. This review aims to summarize recent advances in this combined approach and to discuss the underlying mechanisms driving its potential benefits. (Graphical Abstract image was Created in BioRender. rangzi, Y. (2026), https://BioRender.com/wsui07q).

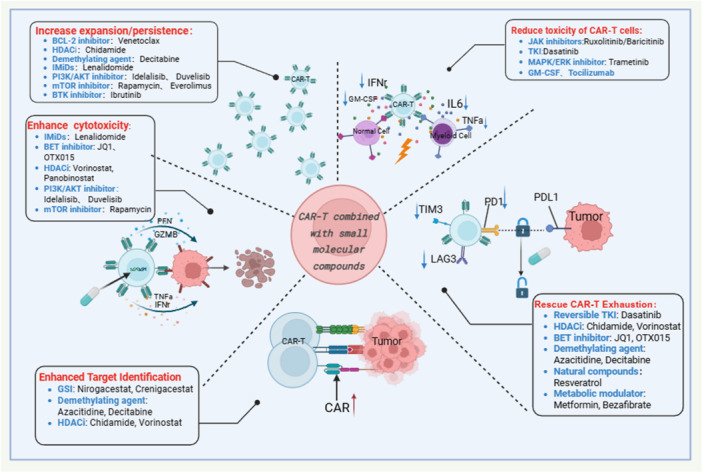

**Abbreviations**
AML: acute myeloid leukaemia
BCMA: B‐cell maturation antigen
BiTE: bispecific T‐cell engager
BTKi: Bruton's tyrosine kinase inhibitor
B‐ALL: B‐cell acute lymphoblastic leukaemia
CAR: chimeric antigen receptor
CIR: cumulative incidence of relapse
CLL: chronic lymphocytic leukaemia
CR: complete response
CRS: cytokine release syndrome
CTL: cytotoxic T lymphocyte
DLBCL: diffuse large B‐cell lymphoma
ENA: enasidenib
FDA: Food and Drug Administration
FH: fumarate hydratase
GA: gallic acid
HAT: histone acetyltransferase
HDACi: histone deacetylase inhibitors
HLH: hemophagocytic lymphohistiocytosis
HSCT: hematopoietic stem cell transplantation
ICANS: immune effector cell‐associated neurotoxicity syndrome
IMiD: immunomodulatory imide drug
LDC: lymphodepleting chemotherapy
LFS: leukaemia‐free survival
MAPK: mitogen‐activated protein kinase
MCL: mantle cell lymphoma
MHC: major histocompatibility complex
MM: multiple myeloma
OR: overall response
ORR: overall response rate
OS: overall survival
PFS: progression‐free survival
PR: partial response
PROTAC: proteolysis‐targeting chimaera
scFv: single‐chain variable fragment
SD: stable disease
TAA: tumour‐associated antigen
TCR: T‐cell receptor
TF: transcription factor
TKI: tyrosine kinase inhibitor
TME: tumour microenvironment
TNBC: triple‐negative breast cancer
TSA: tumor‐specific antigen
TSN: tirucallane‐type triterpenoid

## 1 Background

Malignancies pose a serious threat to people's survival and health due to their high fatality rate. Traditional treatment methods such as chemotherapy, radiotherapy, and surgical treatment have very limited efficacy. Recently, major advances in oncology have shifted therapeutic strategies from broad‐spectrum cytotoxic agents to targeted therapies [1]. Chimeric antigen receptor (CAR) T‐cell therapy has emerged as a breakthrough treatment for various malignancies, especially lymphomas and leukaemias [2]. CAR T cells can recognize tumour‐specific antigens (TSAs) or tumour‐associated antigens (TAAs) expressed on the tumour cell surface via the extracellular antigen‐binding (or recognition) domain. The antibody‐derived recognition domain enables antigen presentation in a non‐major histocompatibility complex (MHC)‐dependent manner [3, 4].

Currently, the primary approved targets for CAR T‐cell therapy are B‐cell maturation antigen (BCMA) for multiple myeloma (MM) [5] and CD19 for various lymphoid malignancies, including B‐cell acute lymphoblastic leukaemia (B‐ALL) and diffuse large B‐cell lymphoma (DLBCL) [6, 7, 8]. In multiple clinical trials of CD19‐specific CAR T cells, complete remission rate is 70%–90% in both pediatric and adult patients with relapsed B‐ALL [9, 10, 11]. For example, CTL019 achieved high response rates even among patients who had relapsed following stem cell transplantation, with durable responses lasting up to 24 months (funded by Novartis; ClinicalTrials.gov identifiers: NCT01626495 and NCT01029366) [11]. The Food and Drug Administration (FDA) has approved 7 CAR T‐cell therapies that target either CD19 or BCMA [12], which is a major milestone in the clinical translation of CAR T‐cell therapy.

CAR T‐cell therapy faces several challenges that limit its clinical efficacy, such as severe and potentially toxicity, suboptimal antitumour activity in solid tumours, antigen escape, and limited tumour infiltration [13, 14, 15]. Another major challenge in CAR T‐cell therapy is T‐cell exhaustion, which compromises the proliferation and persistence of CAR T cells [16, 17, 18, 19]. T‐cell exhaustion in the tumour microenvironment (TME) has been well established to play a role in the activity of immune checkpoint inhibitors (ICIs) and as a significant mechanism for resistance to cellular‐based immunotherapy [20]. The upregulation of inhibitory checkpoint molecules, such as PD‐1, TIM‐3, and LAG‐3 on CAR T cells, is a hallmark of exhaustion [21, 22]. Additionally, TMEs contain additional limitations such as the presence of suppressive immune cells/cytokines, the upregulation of inhibitory receptors, and the transcriptional or metabolic reprogramming induced by chronic antigen stimulation. Collectively, these factors drive CAR T‐cell exhaustion and reduce antitumour efficacy [23].

To address these challenges, many combinatorial strategies are being explored. Preclinical and clinical studies have shown that combining CAR T‐cell therapy with chemotherapy, radiotherapy, oncolytic viruses, vaccines, cytokines, ICIs, bispecific T cell engagers (BiTEs), immunomodulatory drugs, hematopoietic stem cell transplantation (HSCT), or metabolic inhibitors can significantly improve treatment outcomes [24, 25]. The high‐dimensional tools like single‐cell multi‐omics, spectral flow cytometry, and ATAC‐seq enable precise map CAR T‐cell exhaustion, which helps us design more effective combination therapeutic approaches [26, 27, 28]. In this review, we focus on the potential of small‐molecule compounds in combination with CAR T‐cell therapy as promising strategies to overcome T‐cell exhaustion, enhance persistence, and improve therapeutic efficacy.

## 2 The Potential of Small‐Molecule Drugs for Modulating CAR T Cell Therapy

**Figure 1:**
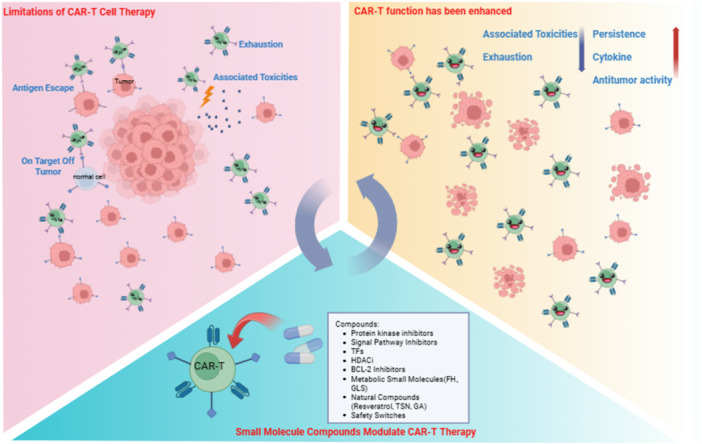
Small Molecule Compounds that Modulate CAR T‐cell Therapy. Created in BioRender. rangzi, Y. (2026), https://BioRender.com/e6zkip4.

Recently, several studies have shown that combination with chemotherapy has dramatically increased the effectiveness of CAR T‐cell therapy and reduced its side effects (Figure 1) [29]. Small molecules have characteristics such as low drug molecular weight, strong tissue permeability, and diverse administration routes. They can enhance the efficacy of CAR T cells through multiple mechanisms, including regulating the TME, promoting T‐cell activity and persistence, inhibiting tumour growth, preventing T‐cell exhaustion, and improving safety [30].

The protein kinase and signalling pathway inhibitors can modulate intracellular signalling, thereby reducing the risk of cytokine release syndrome (CRS) and preventing T‐cell exhaustion [31, 32]. Transcription factor (TF) modulators and histone deacetylase inhibitors (HDACi) remodel the epigenetic and transcriptional landscapes of CAR T cells, maintaining effector functions and enhancing persistence [33, 34]. Moreover, BCL‐2 inhibitors regulate CAR T‐cell survival and functional states by partially controlling the balance of apoptosis thresholds [35]. Metabolic regulators, such as glutaminase (GLS) inhibitors, enhance CAR T‐cell persistence, and antitumour activity across multiple models by reprogramming metabolic and differentiation states [36]. Several natural compounds, like resveratrol, tanshinone (TSN), and glycyrrhizic acid (GA), have the multiple target immunomodulatory and anti‐inflammatory property profiles, suggesting they may help reduce excessive inflammation such as cytokine storms [37, 38, 39]. Additionally, safety switch molecules provide an additional pharmacological control layer, enabling reversible activation or clearance of CAR T cells to enhance safety [32, 40, 41, 42]. Combining these small‐molecule modulators into CAR T therapy offers new strategies to improve efficacy and safety by changing the treatment model from highly toxic regimens towards more durable and controllable interventions.

### 2.1 Antiapoptotic Inhibitors Combined With CAR T‐Cell Therapy

A Key feature of cancer is that tumour cells always hijack normal growth pathways to avoid cell death. They often overexpress antiapoptotic genes, which makes them resistant to certain drugs [43]. Combining antiapoptotic inhibitors with CAR T cells is anticipated to block these resistance pathways while simultaneously activating extrinsic killing mechanisms, thereby producing a synergistic therapeutic effect.

Among these targets, BCL‐2 is a critical antiapoptotic protein that is overexpressed in different cancers, such as B‐NHL, acute myeloid leukaemia (AML), melanoma, breast cancer, and prostate cancer [44]. In addition, BCL‐2 expression is associated with poor clinical outcomes [45]. Preclinical studies showed that pharmacologic inhibition of BCL‐2 may enhance CAR T‐cell–mediated induction of cancer apoptosis [35]. For example, Karlsson et al. demonstrated that the BCL‐2 inhibitor ABT‐737 cocultured with CAR T cells, which significantly enhanced the cytotoxic efficacy of CAR T cells derived from both healthy donors and patients with B‐ALL [46]. ABT‐737 remains in the preclinical stage, whereas venetoclax has been approved by the FDA and is widely used to treat haematological malignancies. One clinical trial (NCT06481241) was designed to evaluate the efficacy and safety of CAR T‐cell therapy combined with venetoclax for patients who were diagnosed with B‐ALL. This trial aimed to increase the apoptotic response and enhance the effectiveness of CAR T cells in eliminating leukaemia cells. In this clinical trial, BCL‐2 inhibitors are typically employed as bridging therapy or short‐term sensitizers, administered prior to or concurrently with CAR T‐cell infusion. This strategy aims to reduce the tumour apoptotic threshold while avoiding the detrimental effects of prolonged exposure on CAR T‐cell persistence [47].

Beyond venetoclax, inhibitors of apoptosis proteins (such as birinapant), Mcl‐1 inhibitors (such as S63845), and Smac mimetics (AZD5582) have been shown to increase the anti‐tumour activity for CAR T‐cell therapy [47, 48, 49]. Most of these compounds are still in preclinical or Phase I clinical development, primarily evaluated as monotherapies or in combination with chemotherapy and ICIs; research into their combination with CAR T cells is currently limited to preclinical proof‐of‐concept studies. Regarding combination strategies, these drugs are typically administered shortly before or after CAR T‐cell infusion to potentiate death receptor‐associated apoptotic signaling pathways triggered by CAR T cells. Notably, the dosage and timing of administration must be rigorously optimized to avoid detrimental effects on CAR T‐cell survival and functional persistence [50, 51, 52]. The synergistic combination of antiapoptotic inhibitors with CAR T‐cell therapy represents a promising avenue currently under clinical investigation, with ongoing trials expected to provide critical insights supporting the clinical translation of this strategy.

Although there are multiple mechanistic synergistic possibilities between anti‐apoptotic inhibitors and CAR T‐cell therapy, it is important to carefully weigh the safety concerns associated with using these agents concurrently. BCL‐2 family inhibitors (such as venetoclax and MCL‐1 inhibitors) can cause cytopenias and lymphocyte apoptosis in a dose‐dependent manner, leading to transient immunosuppression, which may affect CAR T‐cell expansion and in vivo persistence [53, 54]. Systematic reviews and meta‐analyses of multiple clinical trials have shown that these toxic reactions need to be carefully managed through dose adjustment and close monitoring to reduce the safety risks associated with combination therapy [55].

### 2.2 Tyrosine Kinase Inhibitors (TKIs) Combined With CAR T‐Cell Therapy

Targeted therapies, especially TKIs, are crucial in cancer treatment because they can simultaneously inhibit multiple oncogenic signaling pathways. TKIs can be broadly grouped into three groups: (i) directly target cancer cells, (ii) normalize angiogenesis, and (iii) modulate hematopoietic lineage cells [56]. Combining TKIs with CAR T‐cell therapy is an innovative strategy to improve the safety and efficacy of cancer immunotherapy. This method utilizes the distinct mechanisms of both modalities to overcome the limitations of monotherapy.

Preclinical studies indicate that Bruton's tyrosine kinase inhibitor (BTKi) ibrutinib can synergize with CAR T cells. In xenograft models of acute lymphoblastic leukaemia (ALL), chronic lymphocytic leukaemia (CLL), and mantle cell lymphoma (MCL), this combination reduced tumour burden and provided long‐term disease control in the majority of animals compared to monotherapy with only one therapy [57, 58, 59]. The increased effectiveness of ibrutinib is attributed to its positive immunomodulatory effects on CAR T cells, such as promoting their expansion postinfusion and augmenting their effector function through PD‐1 downregulation [57, 60].

This combination addresses significant challenges in current cell therapies. CAR T‐cell therapy alone has drawbacks like poor expansion, T‐cell exhaustion, and pre‐existing antigen resistance. Similarly, while BTKi as a single agent has the potential for some efficacy, this will be limited due either to treatment intolerance or limited efficacy [61, 62, 63]. In the landscape of clinical research, ibrutinib is approved and widely used for B‐cell malignancies. The combination with CAR T‐cell therapy has been demonstrated through initial findings from ZUMA‐2 as well as real‐world studies, but it generally remains in the early‐to‐mid stages of clinical investigation. BTK inhibitors are primarily used as bridging therapy prior to CAR T‐cell infusion to reduce tumour burden. Meanwhile, some studies have explored short‐term combination therapy during the peri‐infusion or early post‐infusion period to support CAR T‐cell expansion and function [57, 64, 65, 66, 67]. However, the immunomodulatory effects of BTK inhibitors have the potential to influence persistence in CAR T cells. This will either present an increased opportunity for infection or hematological toxicity when used in conjunction with lymphodepleting agents. As a result of these concerns, dosing and schedule should be evaluated. Collectively, the data suggest that combination regimens can yield superior patient outcomes.

In addition to BTKis, certain TKIs can be used to pharmacologically modulate CAR T cells directly. Dasatinib is an example of a TKI that can effectively and reversibly suppress proximal CAR signalling kinases. Dasatinib is currently, primarily in the preclinical and early clinical investigative stages. Administering dasatinib induces a temporary “rest” state in CAR T cells, preventing them from acquiring exhausted phenotypic, functional, and epigenetic features [68]. This forced rest, achievable by adjusting the dose and timing of dasatinib, preserves CAR T‐cell function under high tumour burdens and can even reinvigorate exhausted cells, thereby improving their antitumour potency [68, 69]. Furthermore, this method has critical safety applications. The timing of its combination is important, and it is usually designed for use shortly after CAR T‐cell infusion. Mestermann et al. showed that administering dasatinib shortly following the infusion of the CAR T cells results in fast and reversible impairment of T‐cell activation via LCK blockade, significantly reducing CRS‐associated mortality in a mouse model [32].

TKIs may enhance CAR T‐cell efficacy; however, careful attention should be paid to potential toxicity and signalling pathways interference when combined with CAR T therapy. For example, dasatinib inhibits TCR proximal signalling kinases (such as LCK), thereby temporarily blocking the activation, expansion, and effector function of CAR T cells [32]. In contrast, ibrutinib exerts relatively weaker direct inhibition on T‐cell function, but its combination may increase the risk of opportunistic infections and exacerbate treatment‐related immunosuppression [67]. Notably, given that TKI‐mediated signal inhibition is typically reversible and dose‐dependent, this indicates that dosage and dosing schedules in combination regimens need to be precisely optimized.

In conclusion, TKIs can be used alongside CAR T‐cell therapy as versatile adjuncts. They can be used to create synergy in increasing the anti‐tumour effect of the combined therapies while also being able to manipulate pharmacologically as a switch to reduce toxicity and prevent T‐cell exhaustion. Future efforts should focus on optimizing the timing, dosing, and type of TKIs to maximize the clinical benefits of this combination strategy.

### 2.3 Epigenetic Inhibitors Combined With CAR T‐Cell Therapy

The therapeutic efficacy of CAR T‐cell therapy is often limited by T‐cell exhaustion. This functional impairment is characterized by reduced proliferation capacity, impaired cytotoxicity, and sustained upregulation of inhibitory receptors. This exhausted phenotype primarily results from persistent antigen stimulation within the TME or tonic signalling by self‐aggregating CAR constructs [17, 70]. Strong evidence indicates that epigenetic regulation is the central mechanism governing the dysfunction of CAR T cells. Multiple epigenetic changes, including chromatin remodeling, histone acetylation, DNA methylation, and RNA modification, collectively drive CAR T cells into an exhausted state, thereby determining their functional fate and therapeutic potential [71, 72, 73, 74, 75].

The fundamental role of epigenetic regulation in the biology of T cells is the basis of the mechanisms controlling gene accessibility. Histone acetylation, catalysed by histone acetyltransferases (HATs), promotes chromatin relaxation and facilitates transcription, whereas histone deacetylases (HDACs) remove acetyl groups to condense chromatin and suppress gene expression. HDACis exploit this balance by selectively inhibiting HDACs, causing chromatin relaxation, transcriptional reprogramming, and restoring the expression of silenced tumour suppressor genes [76, 77]. In addition to transcriptional modulation, HDACis exert direct antitumour effects by inducing apoptosis and enhancing tumour antigen presentation, making them rational combination partners for CAR T‐cell therapy [78, 79].

Substantial preclinical and clinical evidence support the synergistic potential of combining HDAC inhibitors with CAR T cells. Clinical evidence indicates that in B‐cell malignancies, the selective HDACi mocetinostat achieved an 18.9% overall response rate in a phase II trial for relapsed/refractory DLBCL [80]. In solid tumours, vorinostat (SAHA) potently upregulated B7‐H3 expression on tumour cells, thereby increasing B7‐H3 CAR T‐cell activity [81]. Similarly, panobinostat increased CAR T‐cell cytotoxicity against pancreatic tumours, whereas chidamine enhanced CD22 expression on B‐cell tumours through post‐translation modifications, subsequently improving CD22 CAR T‐cell function [82, 83]. The class I HDAC inhibitor romidepsin further demonstrated its ability to increase NKG2D ligand expression on ovarian cancer cells, thereby increasing NKG2D CAR T‐cell cytotoxicity [84, 85]. Collectively, in the clinical landscape, established HDAC inhibitors such as vorinostat, belinostat, romidepsin, and panobinostat have demonstrated antitumour activity as monotherapies in Phase II/III trials for hematological malignancies, while chidamide is being actively explored in CAR T combination trials [86, 87].

Among these combination strategies, HDAC inhibitors are typically used for short periods before the treatment to sensitize tumour cells prior to CAR T cells infusion, or they are introduced during the early expansion stage to optimize cell function and tumour antigen expression, thereby enhancing the recognition ability and cytotoxicity of CAR T cells [83]. In contrast, M344, entinostat, and other class I/II selective histone deacetylase (HDAC) inhibitors are still in the pre‐clinical stage. In vitro and in vivo studies have demonstrated synergistic effects with CAR T and elucidated mechanisms such as upregulation of tumour antigens, enhanced cytotoxicity, and improvement of memory phenotypes resistant to exhaustion [34]. These findings collectively highlight the potential of HDAC inhibitor maintenance strategies to achieve superior clinical outcomes when combined with CAR T‐cell therapy.

HDAC inhibitors can improve the antitumour efficacy of CAR T cells, however, their associated adverse effects—including myelosuppression, increased risk of infection, and cardiotoxicity—may overlap with CAR T‐related toxicities such as CRS [84]. Meanwhile, continuous or high‐dose exposure to certain histone deacetylase (HDAC inhibitors) can inhibit the proliferation and signal transduction of T cells, which may limit the expansion and long‐term survival of CAR T cells [88]. Therefore, current researches mainly use short‐term, low‐dose, or temporally controlled dosing strategies to mitigate toxicities and drug interactions while preserving the immunomodulatory benefits of these agents.

In addition to histone modifications, DNA methylation is another critical epigenetic regulatory layer that affects CAR T‐cell function. The exhaustion phenomenon of human CAR T cells has been mechanistically related to de novo DNA methylation of genes essential for T‐cell pluripotency and memory formation [75]. By targeting this pathway, CAR T cells manufactured following exposure to the DNA demethylating agent decitabine (d‐CAR T) exhibit significantly enhanced therapeutic properties. Preclinical studies have shown that d‐CAR T cells display increased proliferative capacity, increased cytokine production, sustained memory phenotypes, and potent tumour lytic activity even at low effector‐to‐target ratios. These cells effectively eliminate large mature tumours in vivo and maintain strong memory responses upon tumour rechallenge [89, 90]. Translationally, a lymphodepletion regimen using decitabine combined with CD19/CD22 bispecific CAR T cells significantly improved survival in patients with relapsed/refractory B‐ALL, providing crucial clinical validation for this approach [90].

Combination strategies can synergistically enhance therapeutic efficacy by modifying the epigenetic state of T cells through in vitro treatment during the manufacturing phase or by augmenting target antigen expression and microenvironmental sensitivity through lymphocyte clearance before infusion or tumour pretreatment. However, such methods also introduce risks, including cumulative myelosuppression and the potential exacerbation of CRS [91, 92]. Therefore, the timing and dosage of administration must be carefully balanced within treatment regimens to enhance CAR T‐cell persistence and function while effectively controlling toxicity.

Despite this promise, integrating epigenetic editing into CAR T‐cell manufacturing adds considerable complexity and cost. Although epigenetic regulation is considered safer than permanent genome editing due to its reversibility, it still requires rigorous evaluation of targeting and off‐target effects, especially considering that key regulatory genes such as DNMT3A and PRDM1 are closely associated with tumour development [89, 93]. Future progress will rely on multi‐omics technologies to dynamically depict the epigenetic landscape of exhausted CAR T cells, to identify new targets and formulate rational intervention strategies. Combining CAR T‐cell therapy with clinically approved epigenetic drugs offers a practical approach to enhance efficacy while using existing safety data.

In conclusion, epigenetic inhibitors are powerful adjuncts to CAR T‐cell therapy and exert their effects through two mechanisms: directly reprogramming CAR T cells towards persistent, non‐exhausted phenotypes, and modulating the TME to increase antigen recognition and cytotoxicity. Although challenges remain in manufacturing complexity and safety assessment, the strategic integration of epigenetic modulation holds great potential to overcome the key obstacle of T‐cell exhaustion and unlock durable responses across a broader spectrum of malignancies.

### 2.4 Immunomodulatory Agents Combined With CAR T‐Cell Therapy

The efficacy of CAR T‐cell therapy can be significantly enhanced by combining it with immunomodulatory agents. These immunomodulatory agents exert their effects through diverse mechanisms to overcome tumour resistance and reshape the immunosuppressive TME [94]. This integrated approach is a promising strategy to increase the potency, persistence, and infiltration of CAR T cells in different cancer types.

In triple‐negative breast cancer (TNBC), EGFR‐targeted CAR T cells effectively suppress tumour growth in vitro and in vivo, but the rapid emergence of resistance in some models limits their therapeutic potential. Xia et al. demonstrated that this resistance is associated with CAR T‐cell‐induced upregulation of immune‐suppressive genes, which are associated with increased sensitivity to THZ1, a CDK7 inhibitor that targets epigenetic regulators. The combination of THZ1 with EGFR CAR T cells successfully mitigated immune escape, suppressed tumour growth, and inhibited metastasis in TNBC models, highlighting the role of epigenetic modulation in reversing acquired resistance [95].

The immunosuppressive TME represents another major barrier to CAR T‐cell efficacy. Anti‐angiogenic agents such as bevacizumab, which specifically target vascular endothelial growth factor (VEGF), can normalize and remodel the tumour vasculature to increase CAR T‐cell infiltration. In neuroblastoma models, bevacizumab combination therapy induced significant vascular disruption and increased the infiltration of GD2‐targeted CAR T cells into tumour tissue. This effect was further enhanced by bevacizumab‐mediated upregulation of the expression of CXCL10, a chemokine that promotes T‐cell recruitment, resulting in improved antitumour efficacy [96].

Immunomodulatory imide drugs (IMiDs), such as lenalidomide, also exhibit strong potential for synergistic combination with CAR T cells. A Phase II clinical trial (NCT05032820) has been registered to systematically assess lenalidomide combined with anti‑BCMA CAR T in MM, but full results are not yet published. Preclinical experimental evidence indicates that lenalidomide enhances the quality of the immune synapses (ISs) in CAR T cells, leading to significantly increased F‐actin polymerization at the contact site with tumour cells. In glioblastoma models, EGFRvIII‐targeted CAR T cells combined with lenalidomide demonstrated enhanced cytotoxicity, improved tumour infiltration, and prolonged survival in vivo [97]. Mechanistically, lenalidomide upregulates NF‐κB signalling downstream of the CD28 costimulatory domain and promotes T‐cell proliferation. Lenalidomide also counteracts the immunosuppressive effects mediated by checkpoint molecules such as CTLA‐4 [98]. These findings collectively suggest that IMiDs can enhance the function of CD28‐containing CAR T cells, enabling them to overcome the inhibitory TME more effectively. Lenalidomide is typically administered concurrently with CAR T‐cell infusion or during the early post‐infusion period. While IMiDs enhance CAR T‐cell function and persistence, their immunostimulatory properties may increase the risk of CRS and neurotoxicity, as well as contribute to cumulative hematologic toxicity. Therefore, further clinical studies should be conducted to observe blood count and immune‐related adverse reactions.

In short, targeted therapies and immunomodulatory agents including epigenetic regulators, antiangiogenic drugs, and IMiDs‐complement CAR T‐cell therapy by targeting specific cancer cell alterations or modulating the TME, whereas CAR T cells directly recognize and eliminate tumour cells through surface antigens. Integrating these modalities can deliver a more comprehensive attack on malignant cells [99], bringing a multifaceted strategy to improve clinical outcomes by addressing the intrinsic tumour resistance and extrinsic immunosuppressive mechanisms.

### 2.5 Signalling Pathway Inhibitors Combined With CAR T‐Cell Therapy

A common method for finding small‐molecule drugs is functional screening based on specific signalling pathways or key molecules within these pathways. As understanding of these pathways deepens, researchers can identify potential targets and design small‐molecule compounds to modulate them. This strategy facilitates the development of novel therapeutic interventions that aim at precisely regulating signalling pathways for disease treatment.

#### 2.5.1 PI3K/AKT/mTOR Pathway Inhibitors Combined With CAR T‐Cell Therapy

The PI3K/AKT/mTOR signalling axis, as a central regulator of T‐cell biology, governs key processes such as proliferation, survival, metabolism, and functional differentiation. After T‐cell activation, the binding of the T‐cell receptors (TCRs) and costimulatory molecules activates signalling cascades to promote clonal expansion and effector differentiation [100]. In the framework, mTOR acts as a key integrator of environmental cues, directing T‐cell fate decisions. However, this pathway is frequently activated in hematologic and solid malignancies, making it a compelling therapeutic target [101]. Strategic inhibition of the PI3K/AKT/mTOR pathway represents a promising approach to increase the efficacy and persistence of CAR T‐cell therapies.

PI3K inhibitors have demonstrated significant potential in modulating CAR T‐cell properties. FDA‐approved agents such as idelalisib (PI3Kδ‐specific), duvelisib (PI3Kδ/γ), and copanlisib (pan‐PI3K) are primarily used to treat hematologic malignancies [102, 103, 104, 105, 106]. Although these inhibitors have been clinically validated as anticancer therapies, evidence supporting their use to enhance CAR T‐cell function remains largely preclinical. Ongoing early‐phase clinical trials are assessing the safety and efficacy of combination strategies. At present, PI3K inhibitors are primarily applied during the *in vitro* manufacturing of CAR T cells. When applied during CAR T‐cell manufacturing, these inhibitors—particularly those targeting PI3Kδ/γ—help maintain a less differentiated, stem cell‐like state in CD8 + CAR T cells. This pharmacological preconditioning enhances their in vivo persistence and antitumour capacity against cancers such as CLL without compromising their expansion potential [31, 107, 108]. For example, preharvest treatment with duvelisib during in vitro expansion has been proposed as a broadly applicable strategy to reverse T‐cell immune paralysis and improve patient responses [109, 110].

In addition to PI3K, downstream mTOR inhibition offers additional immunomodulatory benefits. Rapamycin and its analogues (rapalogs) selectively inhibit mTOR complex 1 (mTORC1) [111, 112]. Moreover, mTOR inhibitors are among the most effective agents for mitigating T‐cell‐bispecific antibody‐induced cytokine release, significantly reducing inflammatory cytokine production while preserving T‐cell cytotoxicity—a valuable safety feature for combination regimens [113]. mTOR inhibitors have shown limited early clinical use in CAR T‐cell therapy, where they are primarily administered short‐term after CAR T‐cell infusion to manage severe or refractory CRS and CAR T‐associated hemophagocytic lymphohistiocytosis (HLH). In contrast, their application as a combination strategy to directly enhance CAR T‐cell antitumour efficacy remains confined to mechanistic studies and preclinical investigation.

Conversely, strategic activation of certain pathway components can also be beneficial. The PP2A inhibitor LB‐100 enhances T‐cell antitumour activity by promoting mTOR signalling. LB‐100 combined with anti‐CAIX CAR T cells showed enhanced efficacy in vitro and in vivo; thus, modulating phosphatase activity is expected to enhance the function, even in challenging solid tumours such as glioblastoma [114, 115]. However, this strategy is currently limited to preclinical studies, and its potential systemic toxicity must be rigorously evaluated before clinical translation.

Notably, cytokine signalling during expansion can affect pathway activity and the fitness of CAR T cells. Compared with IL‐2‐cultured cells, CAR T cells expanded in IL‐15 (CAR T/IL‐15) maintain a stem‐like memory (Tscm) phenotype, exhibit decreased mTORC1 activity, improved mitochondrial fitness, and lower expression of exhaustion markers [116]. This metabolic and phenotypic profile underpins their increased persistence and antitumour capacity.

In conclusion, the PI3K/AKT/mTOR pathway represents a flexible therapeutic target for improving CAR T‐cell therapy. Pharmacologic inhibition at various targets (PI3K, mTOR) can increase stemness, persistence, and safety, whereas targeted activation (via PP2A inhibition) or cytokine‐mediated modulation (IL‐15) can similarly promote favourable functional states. These approaches, which are grounded in a deep understanding of T‐cell signalling and metabolism, provide rational combination strategies to overcome current limitations in CAR T‐cell immunotherapy for both hematologic and solid malignancies.

#### 2.5.2 JAK‐STAT Signalling Pathway Inhibitors Combined With CAR T‐Cell Therapy

The JAK‐STAT signalling pathway serves as a crucial pathway for cytokine‐mediated communication within the TME and plays a dual role in cancer progression and immune regulation [117]. This pathway is activated by numerous cytokines and growth factors and JAK kinases (JAK1, JAK2, JAK3, TYK2) phosphorylate cytokine receptors and recruit STAT transcription factors (STAT1–6) to drive gene expression [118]. Recent studies have highlighted the particular importance of JAK‐STAT signalling in T‐cell activation and proliferation, which makes it an ideal target for drug targeting in CAR T‐cell therapy [119, 120, 121]. Targeting this pathway offers a promising approach to reduce treatment toxicity while maintaining antitumour efficacy.

Modulating the JAK‐STAT signaling pathway is widely used to control the CRS in CAR T‐cell therapy. Tocilizumab, an anti‐interleukin‐6 receptor antibody, is the first‐line treatment for severe CRS by blocking IL‐6‐mediated JAK‐STAT activation [122, 123], indicating that JAK inhibitors could be used for CRS treatment. Itacitinib, a selective JAK1 inhibitor, has demonstrated particular promise in preclinical models because it significantly reduces CRS‐associated cytokine levels without impairing CAR T‐cell proliferation or antitumour activity [124]. Recently, a phase II clinical trial (NCT04071366) showed that itacitinib combined with CAR T‐cell therapy could reduce inflammation, CRS, and ICANS, without impairing CAR T‐cell antitumour activity [125].

Beyond CRS management, JAK inhibitors have subtle effects on CAR T‐cell biology. Ruxolitinib is the first JAK inhibitor approved for myelofibrosis and presents a complex pharmacological profile. Multiple in vitro and animal studies have demonstrated that it significantly suppresses CAR T‐cell proliferation and cytokine release from multiple immune cell types, but it simultaneously promotes a therapeutically favourable differentiation phenotype. Importantly, following drug withdrawal, the CAR T cells regain cytolytic capacity, although cytokine production is partially inhibited [126]. This timing control enables strategic dosing regimens that may separate efficacy from toxicity. Clinically, Ruxolitinib has been shown in a case report to rapidly ameliorate steroid‐refractory CRS in patients receiving CD19‐ or CD22‐directed CAR T‐cell therapy without the appearance of significant adverse events [127]. Moreover, a registered phase I clinical trial is currently evaluating the safety and preliminary efficacy of the combination of Ruxolitinib and CART123 cells for the treatment of relapsed or refractory AML (NCT06768476).

The clinical application of the JAK inhibitor arsenal continues to expand, with agents such as fedratinib offering more treatment options. Although currently approved for the treatment of myeloproliferative disorders, fedratinib has demonstrated antitumour activity in Hodgkin lymphoma and mediastinal large B‐cell lymphoma models, suggesting it also has potential for activity in haematologic malignancies, including the conditions targeted by CAR T‐cell therapy [128]. At present, the evidence supporting the combination of fedratinib and CAR T‐cell therapy is mainly based on in vitro and animal studies.

In summary, targeting the JAK‐STAT pathway may be an effective way of increasing the therapeutic index of CAR T‐cell therapy. By selecting specific inhibitors and dosing schedules, clinicians may distinguish the expected antitumour effects from harmful inflammatory responses. This approach not only addresses the critical challenge of CRS but also may favourably influence CAR T‐cell differentiation states, thereby gradually enhancing safety and therapeutic efficacy in various malignancies.

#### 2.5.3 MAPK/ERK Signalling Pathway Inhibitors Combined With CAR T‐Cell Therapy

The mitogen‐activated protein kinase (MAPK) cascade, and in particular the classic Ras/Raf/MEK/ERK pathway, is the cornerstone of the intracellular signalling that governs fundamental cellular processes, including proliferation, differentiation, and survival. In oncology, hyperactivation of this pathway represents a widely recognized oncogenic driver in many cancers, acting to promote cancer initiation, progression, and therapeutic resistance [129]. The critical role of ERK signalling in tumour development has made targeted therapy a major focus, with small‐molecule inhibitors against this pathway demonstrating significant clinical success, notably in metastatic melanoma, where they have substantially improved disease control [130].

The strategic integration of MAPK/ERK pathway inhibitors with CAR T‐cell therapy is emerging as a reasonable solution to overcome the limitations of each modality. Although kinase inhibitors can induce strong but transient tumour regression, CAR T‐cell therapy holds promise for durable immune‐mediated control. Preclinical evidence suggests that this combination can yield complementary benefits. For example, the BRAF inhibitor dabrafenib modulates the in vitro functionality of GD2‐specific CAR T cells, suggesting a direct pharmacological influence on cellular immunotherapy products [131]. Current research on the combined application of MAPK/ERK pathway inhibitors and CAR T‐cell therapy is still mainly limited to in vitro, animal model studies, and theoretical investigations.

Mechanistically, MAPK pathway inhibition may enhance CAR T‐cell therapy through several potential pathways [132]. By disrupting survival signals in tumour cells, these inhibitors can enhance the sensitivity of malignant cells to CAR T‐cell‐mediated killing. Furthermore, modulation of the TME through MAPK/ERK inhibition can improve CAR T‐cell infiltration and persistence in tumour sites. This combination approach holds particular promise for solid tumours, where the immunosuppressive microenvironment and dense stromal architecture frequently impede the efficacy of CAR T cells.

In summary, combining MAPK/ERK pathway inhibitors with CAR T‐cell therapy is a promising multimodal strategy that combines the potent cytoreductive capacity of targeted agents with the durable adaptive immune response to cellular immunotherapy. Future studies will focus on clarifying the optimal sequencing, dosing, and patient selection criteria for these combinations, which will be crucial to fully realizing their potential in the clinical field.

#### 2.5.4 Wnt/β‐Catenin Signalling Pathway Inhibitors Combined With CAR T‐Cell Therapy

The Wnt/β‐catenin signalling pathway represents an evolutionarily conserved mechanism, regulating cell–cell communication during embryonic development and tissue homeostasis in the adult. Dysregulation of this pathway is implicated in a wide variety of human malignancies, suggesting valuable therapeutic opportunities for cancer intervention. Recent investigations have demonstrated that pharmacological modulation of Wnt signalling can significantly increase the efficacy of CAR T‐cell therapy through multifaceted effects on both immune cell function and the TME.

The significant advancements in this area include the development of hsBCL9CT‐24, a novel peptide that specifically disrupts the β‐catenin/BCL9 protein–protein interaction. This inhibitor demonstrates potent antitumour activity through multiple immunomodulatory mechanisms, including enhancing cytotoxic T‐cell infiltration into tumour beds, increasing the number of dendritic cells, and reducing immunosuppressive regulatory T‐cell populations [133]. When combined with EpCAM‐specific CAR T cells, hsBCL9CT‐24 exhibited synergistic antitumour effects on EpCAM‐positive colorectal cancer models both in vitro and in vivo [134].

Mechanistic studies revealed that hsBCL9CT‐24 remodelled the TME to favour antitumour immunity. This remodelling mechanism not only facilitates the generation of early effector T‐cell populations but also significantly delays the occurrence of CAR T‐cell exhaustion, which is the major limitation of current CAR T‐cell therapies. Additionally, treatment with hsBCL9CT‐24 upregulated the expression of the T‐cell recruitment chemokine CXCL10 to increase the degree of T‐cell recruitment and infiltration into tumour sites [134].

The combination of small‐molecule inhibitors targeting the Wnt/β‐catenin pathway with CAR T‐cell therapy has demonstrated significant strategic value in the field of cancer immunotherapy. Pathway modulation seems to enhance the function of CAR T cells and their persistence in the malignant TME. Although clinical trial data are not yet available, a large amount of preclinical evidence provides a compelling foundation for future clinical translation. This combined strategy is particularly promising for solid tumours, in which the immunosuppressive microenvironment often limits the efficacy of CAR T cells. Therefore, further research is necessary to fully elucidate its therapeutic potential.

In conclusion, signaling pathway inhibitors have potential advantages in enhancing efficacy and managing toxicity in CAR T‐cell therapy, but they also face safety challenges. PI3K/AKT/mTOR inhibitors can promote T‐cell memory phenotypes and delay exhaustion during in vitro CAR T‐cell preparation; however, premature administration or excessive dosing may inhibit CAR T‐cell proliferation or function. JAK‐STAT inhibitors can alleviate CAR T‐related CRS and inflammatory responses, but they may induce hematopoietic suppression and metabolic disturbances. Prolonged or excessive JAK‐STAT inhibition can disrupt survival signals, such as IL‐2 and IL‐7, compromising CAR T‐cell persistence [117, 135]. MAPK/ERK inhibitors can transiently regulate the activation of CAR T cells; however, prolonged or high‐dose exposure may damage CAR signaling and lead to “dormancy” of CAR T cells [136]. The effects of Wnt/β‐catenin pathway inhibitors on CAR T cells remain poorly defined and confined to theory, requiring careful thought for dosing design. Overall, the efficacy and safety of combination strategies depend critically on timing, dosage, and drug interactions. In particular, the use of immunosuppressive agents during peak CAR T‐cell activation in vivo requires careful optimization to balance antitumour activity with toxicity risk.

### 2.6 Metabolic Small Molecules Combined With CAR T‐Cell Therapy

The therapeutic potential of CAR T cells is intimately linked to their metabolic fitness within the TME. Both tumour cells and CTLs exhibit extreme proliferative potential with overlapping metabolic dependencies, particularly on capturing glucose and fermenting it to lactate to meet their bioenergetic and biosynthetic requirements. This metabolic competition often disadvantages CAR T cells, limiting their efficacy [137]. However, the emerging strategies that use small molecules to modulate metabolic pathways offer promising options for enhancing the function, persistence, and antitumour activity of CAR T cells.

Glycolytic suppression in tumour cells increases the efficacy of various immunotherapies. Preclinical studies have indicated that modulating the glycolytic activity of CAR T cells can promote a memory T‐cell phenotype and enhance their therapeutic efficacy. For example, CAR T cells engineered with intracellular 4‐1BB costimulatory domains exhibit enhanced mitochondrial biogenesis and oxidative metabolism, which correlate with improved persistence. This provides a rationale for simultaneously exploiting tumour metabolic vulnerabilities while reinforcing CAR T‐cell metabolic fitness [138, 139, 140]. In addition, studies have confirmed that the overexpression of glucose transporter Glut3 in CAR T cells increases glucose uptake under nutrient‐deprived conditions, enhances the anti‐tumour efficacy of these agents in solid tumour models, and improves oxidative phosphorylation and mitochondrial adaptability [141].

The accumulation of specific metabolites in the TME can directly impair CTL function. For example, fumarate accumulation in fumarate hydratase (FH)–deficient tumour models suppresses CD8⁺ T‐cell activation. Analysis of single‐cell RNA sequencing data from patients treated with CD19‐targeted CAR T cells revealed a strong positive correlation between FH expression and CAR T‐cell functional gene signatures. Mechanistically, FH overexpression in CAR T cells reduces intracellular fumarate levels, enhances TCR signalling and cytokine production, and significantly improves tumour clearance both in vitro and in vivo [142]. The modulation of FH in combination with CAR T cells is primarily applied during in vitro manufacturing to enhance effector function, whereas post‐infusion modulation may further sustain antitumour activity within the TME. These findings identify FH augmentation as a promising metabolic strategy to rescue T‐cell function in suppressive TMEs.

In addition to their effects on canonical metabolites, pharmacological agents such as tetracyclines have been shown to influence T‐cell infiltration and activity. A deficiency of ZAP70 signalling in tumour‐infiltrating T cells suppresses CD8⁺ T‐cell recruitment, whereas enhancing this pathway in the TME via ZAP70‐dependent signalling augments CAR T‐cell antitumour efficacy [143, 144]. This therapeutic intervention is optimally applied immediately before or after infusion to promote T‐cell infiltration and enhance early antitumour responses. These findings highlight the role of small‐molecule modulators in TCR signalling and open up a new avenue for improving cell therapies through targeted signaling regulation.

Glutamine metabolism profoundly influences T‐cell differentiation, function, and epigenetic programming [145]. Glutamine‐derived metabolites, such as α‐ketoglutarate (αKG), remodel the epigenetic landscape and modulate chromatin accessibility, and thus influence T‐cell fate decisions [146]. The inhibition of GLS, the enzyme that catalyses glutamine hydrolysis, initially enhances CAR T‐cell effector function. However, long‐term inhibition can damage its long‐term efficacy. Short‐term or intermittent GLS inhibition enhances CAR T‐cell persistence and functions, indicating that timing and duration are critical to therapeutic benefit. Glutamine conditioning or modulation of glutamine metabolism is frequently performed during the in vitro CAR T‐cell expansion phase to promote enhanced proliferation, oxidative metabolism, memory phenotype, and tumour infiltration, resulting in improved functional fitness in vivo [36, 147].

Interestingly, GLS inhibition has dual effects: it promotes Th1 responses and also inhibits the differentiation of Th17 cells [147]. These findings indicate that GLS represents a context‐dependent target; its transient inhibition may program T cells to enhance IFN‐γ–mediated effector responses, while its sustained inhibition could be used to dampen pathological inflammation in autoimmune conditions.

The integration of metabolic interventions with CAR T‐cell manufacturing and postinfusion support is growing. For example, the IDH2 inhibitor enasidenib (ENA), an FDA‐approved agent, was shown to enhance the formation of memory CAR T cells in a mitochondrial‐targeted drug screen. It achieves this by redirecting glucose carbon utilization from glycolysis to the pentose phosphate pathway, reducing oxidative stress, and promoting a memory‐associated epigenetic landscape through increased histone acetylation [148]. ENA is combined with CAR T cells during the in vitro manufacturing stage to enhance memory differentiation and functional persistence. This highlights that pharmacological modulation of a single metabolic enzyme can have multifaceted effects on T‐cell differentiation and function.

Metabolic small molecules represent powerful adjuncts to CAR T‐cell therapy, enabling modulation of T‐cell metabolism to overcome the suppressive TME and enhance antitumour immunity. While these findings are as yet limited to preclinical experiments, they point to promising translational strategies that require additional clinical validation. Careful consideration must be given to prolonged or inappropriate metabolic inhibition (such as that involving GLS or IDH2/ENA), impairing CAR T‐cell persistence, compromising effector function, or inducing transient immunosuppression [149]. Future work should focus on the rational optimization of the dosage and administration sequence of these combination therapy regimens, the development of biomarkers to identify patients most likely to benefit from specific metabolic interventions, and the exploration of the combination of these methods with other treatment modalities (such as immune checkpoint blockade therapy). As the metabolic dialogue between tumours and immune cells becomes better understood, rationally designed combination therapies may be key to realizing the full promise of CAR T cells across hematologic and solid malignancies.

### 2.7 Transcription Factor Inhibitors Combined With CAR T‐Cell Therapy

TFs, which bind to specific DNA sequences and control gene expression, affect T‐cell differentiation, function, and survival. Several studies showed that targeting the transcription factors with molecular compounds can effectively reprogram CAR T cells, prevent them from becoming exhausted, and increase the antitumour activity [150]. Combining TF‐targeted small molecules with CAR T‐cell therapy thus represents a promising synergistic strategy to overcome current limitations in cancer immunotherapy.

MYC is one of the most important transcription factors that controls tumour cell growth, stem cell behaviour, and drug resistance. Targeting MYC with inhibitors not only directly suppresses tumour cells growth but also enhances the sensitivity of cancer cells to CAR T‐cell therapy [151]. For example, in non–small‐cell lung cancer, the EGFR‐targeting antibody cetuximab has been shown to reduce MYC expression and induce tumour cell death [152, 153]. As EpCAM‐directed CAR T cells are already under clinical evaluation (NCT02725125, NCT02915445, and NCT02729493), their combination with MYC inhibitors constitutes a rational approach for precision immunotherapy, especially in solid tumours where CAR T cells often exhibit limited efficacy and significant toxicity [154, 155, 156]. Preclinical studies suggest that combining MYC inhibitors with CAR T‐cell therapy can maximize tumour sensitivity.

Beyond MYC, the NR4A family of transcription factors (NR4A1, NR4A2, and NR4A3) has been identified as pivotal regulators of T‐cell exhaustion. These TFs are consistently upregulated in both tumour‐infiltrating lymphocytes and CAR T cells subjected to chronic antigen stimulation and act as key effectors of the NFAT‐driven exhaustion programme. Genetic ablation of all three NR4A receptors reprogrammed exhausted CD8^+^ T cells towards a potent effector state, markedly enhancing their cytotoxicity. Similarly, compared with their wild‐type controls, NR4A‐deficient CAR T cells presented lower exhaustion levels, longer persistence, and superior antitumour activity [157, 158]. Although NR4A‐targeting strategies remain in the preclinical stage, targeted molecule compounds and protein degradation are promising ways to block NR4A and enhance CAR T‐cell antitumour activity.

Despite this considerable promise, targeting transcription factors for treatment also has special challenges. Many TFs lack defined ligand‐binding pockets and contain intrinsically disordered regions, complicating conventional small‐molecule drug design. Furthermore, these proteins are always expressed in normal cells, and it is difficult to inhibit them specifically in cancer cells without affecting normal cells. Currently, many therapy strategies focus on targeting the upstream regulators or co‐factors of transcription factors instead of the TFs themselves. Therefore, advances in structural biology and proteolysis‐targeting chimaeras (PROTAC) may allow more direct and selective inhibitors of transcription factors in the future [159, 160, 161].

### 2.8 Natural Product Chemistry Combined With CAR T‐Cell Therapy

Natural products have long been key sources for finding new anticancer drugs, with a large proportion of anticancer agents being from natural sources by 2014 [162]. Well‐known examples are paclitaxel, topotecan, irinotecan, and vincristine, which demonstrate that natural compounds are chemically diverse and clinically useful [162]. In CAR T‐cell therapy, several natural compounds can greatly improve treatment effects and reduce side effects. Gallic acid (GA) is a natural molecule found in a wide range of fruits as well as in medicinal plants. Recent studies have demonstrated that combining GA with CAR T‐cell therapy not only enhances antitumour efficacy but also significantly reduces the dose of CAR T cells. This lower dose is especially useful for reducing the risk of CRS, a dangerous side effect of CAR T therapy [163]. The ability of GA to broaden the therapeutic window makes it an attractive adjunctive agent in CAR T therapy.

Another promising candidate is Tirucallane‐type triterpenoids (TSNs), which were originally isolated from neem plants with a history of use in Eastern medicine [164]. In addition to its direct anticancer effects through apoptosis induction and cell cycle arrest, TSN also helps regulate the immune system [165]. In glioblastoma models, TSN reprogrammes tumour‐associated macrophages, reversing their immunosuppressive phenotype and boosting T‐cell infiltration and activation while reducing markers of exhaustion. This macrophage reprogramming makes treatment‐resistant tumours sensitive to CAR T‐cell therapy. Unlike GA, TSNs are mainly given before or together with CAR T‐cell infusion [166]. This approach improves treatment effects by remodelling the TME.

Recently, natural products have been used not just as supplements before or after CAR T infusion but also to create smarter control systems for CAR T cells. For example, Yang's research group designed a resveratrol‐repressible transgene expression (RESrep) CAR device and a resveratrol‐inducible transgene expression (RESind) CAR device based on the natural product resveratrol as a switch molecule, containing gene circuits that can control the activation (on) and inactivation (off) of CAR T cells [167]. These tenable dual‐switch systems provide a safety switch to reduce CAR T toxicity, supporting higher CAR T‐cell expansion and long‐term persistence in a drug‐dependent manner.

The integration of natural products with CAR T‐cell therapy solves several critical challenges in cancer treatment. These compounds can potentially overcome drug resistance, reduce the required doses of cytotoxic agents, reduce the side effects, and improve overall therapeutic efficacy [168]. However, certain natural products, such as polyphenolic compounds, may inhibit immunotherapy when administered at inappropriate doses or timings. This can lead to the generation of immunosuppressive cell clones, thereby promoting tumour growth and metastasis [169, 170]. Challenges remain in optimizing the bioavailability, dosing, and formulation of natural compounds for combination therapy.

In conclusion, natural products offer multiple advantages when combined with CAR T‐cell therapy, acting as efficacy enhancers, toxicity reducers, and even molecular switches for precise control. As research progresses, integrating these naturally derived compounds with advanced cellular immunotherapies holds great promise for the development of more effective and safer treatment methods for various malignant tumours. Future work should focus on clarifying the precise mechanisms of interaction between natural compounds and CAR T cells, optimizing combination protocols, and advancing the most promising combinations into clinical trials.

### 2.9 Chemotherapeutic Agents for Lymphodepletion Prior to CAR T‐Cell Therapy

Lymphodepleting chemotherapy (LDC) prior to CAR T‐cell infusion has become a recognized standard treatment protocol and plays an indispensable role in the efficacy of CAR T‐cell products such as the BCMA‐directed therapies idecabtagene vicleucel (ide‐cel) and ciltacabtagene autoleucel (cilta‐cel) for relapsed/refractory MM [5, 171]. The main function of lymphodepletion is to create a favourable immunological and cytokine environment by clearing endogenous lymphocytes that compete with homeostatic cytokines. This process supports robust CAR T‐cell expansion, enhances CAR T‐cell persistence, and exerts direct cytotoxic effects on the TME, collectively contributing to improved clinical outcomes [9, 172]. It is important to emphasize that lymphodepletion is administered exclusively prior to CAR T‐cell infusion.

The combination of fludarabine and cyclophosphamide (Flu/Cy) remains the most extensively, clinically validated and widely adopted lymphodepletion regimen for CAR T‐cell therapy. However, significant differences in the dosage, duration, and intensity of LDCs exist across different institutions and clinical trials, which indicates that all parties are continuously striving to optimize this critical preparatory phase [173, 174, 175, 176, 177, 178, 179]. Recent nation‐wide shortages of fludarabine have increased the vulnerability of current treatment protocols, leading to treatment delays and underscoring the urgent need for robust alternative lymphodepletion strategies.

Bendamustine is a bifunctional agent that has both alkylating and purine analogue properties and is expected to be one of the new alternatives. Bendamustine has been successfully used as a lymphodepletion regimen for tisagenlecleucel, and early clinical evidence suggests that its safety and efficacy profile are comparable to those of Flu/Cy in relapsed or refractory non‐Hodgkin lymphoma patients [180, 181]. Nevertheless, comprehensive data on its application with other CAR T‐cell products are still limited, highlighting an important area for future investigations.

From a safety perspective, cytopenias due to lymphodepletion and the resulting immunosuppression constitute major clinical toxicities of CAR T‐cell therapy. Neutropenia is the most common manifestation, often leading to secondary infections, particularly in the first weeks after CAR T‐cell infusion, and has a significant impact on the early clinical efficacy. These results highlight the urgent need to balance improving the efficacy of immunotherapy and ensuring patient safety when designing lymphocyte depletion regimens or developing small molecule combination therapy strategies.

The field is tending towards more personalized and targeted preconditioning strategies. Future strategies will focus on optimizing lymphodepletion regimens on the basis of specific disease characteristics, prior therapies, and host immune status. Developing new chemotherapeutic drugs, non‐chemotherapeutic lymphodepletion methods, and combination strategies to further modulate the TME hold great potential for enhancing the expansion, function, and safety of CAR T cells. Through continuous optimization of the pre‐treatment regimen, the efficacy of CAR T‐cell therapy will be significantly improved in a wide range of malignancies.

### 2.10 Small Molecule Switching for CAR T Cells

Although CAR T‐cell therapy has achieved clinical progress, it also brings serious safety risks, such as CRS and immune effector cell‐associated neurotoxicity syndrome (ICANS). To address these issues, scientists have developed a small‐molecule switch system to make this therapy more precise and flexible.

The small‐molecule switches always regulate CAR T cells through different molecular mechanisms. One method uses engineered proteins, with the CAR functioning only in the presence of specific small molecules. For example, lipocalin‐based molecular switches can be designed to respond to orally administered compounds, with one demonstrated system showing a 550‐fold increase in affinity between human retinol‐binding protein 4 (hRBP4) and its engineered binding partner upon the addition of the small molecule A1120. These systems effectively modulate the cytotoxic activity and cytokine production of primary human CAR T cells, highlighting their therapeutic potential [182].

Another innovative strategy involves protease‐based regulation systems such as switch‐off CAR (swift‐CAR) or small‐molecule‐assisted shutoff CAR (SMA ShCAR). These designs incorporate protease targets and degradation determinants into the CAR structure. In the default “on” state, cleavage at the target site removes the degradation determinant, allowing functional CAR expression. When exogenous small‐molecule protease inhibitors are administered, the cleavage process is blocked, and the CAR protein is degraded, effectively “inactivating” CAR T cells [183, 184, 185].

Dimethylaminotetracyclines have become particularly promising regulatory molecules due to their well‐established safety and favourable pharmacokinetic properties. It offers excellent oral bioavailability, rapid absorption (reaching peak plasma concentrations within 2 h), and nearly complete steady‐state bioavailability [186, 187, 188]. Minocycline, a structural analogue of this drug, has the added benefit of effectively crossing the blood–brain barrier, which leads to increased concentrations of the drug in cerebrospinal fluid and is essential for treating CAR T‐cell therapy‐related neurotoxicity [189, 190].

The split CAR “off” system developed by Hotblack et al. exemplifies the application of tetracycline‐based control. This system relies on the interaction between the tetracycline repressor protein (TetR) and a tetracycline peptide analogue (TIP) to form a functional CAR. This structure dissociates when exposed to minocycline. The primary advantage of this approach lies in its rapid reversibility. CAR signalling inhibition occurs quickly upon tetracycline administration and can be restored through withdrawal after toxicity subsides. This makes it particularly suitable for CARs that target novel antigens with limited normal tissue expression and for managing toxicity in CD19‐directed therapies [191].

In addition to the use of tetracycline‐based systems, many diverse small‐molecule approaches have been explored to control CAR T‐cell activity. These include strategies utilizing fluorescein isothiocyanate (FITC), folic acid, limonoids, rapamycin, PROTACs, and dasatinib. Each platform has different advantages in terms of kinetics, reversibility, and clinical translatability. Systematic classification of these novel safety switches provides a framework for selecting appropriate control strategies on the basis of specific clinical requirements [192].

The small‐molecule switch technology for CAR T cells is still in the preclinical stage. However, several small‐molecule‐inducible safety switch systems have progressed to clinical trials. For example, the inducible caspase‐9 (iCasp9) system has been incorporated into multiple CAR T‐cell clinical studies. This system does not directly regulate CAR signalling. Instead, it functions as a safety switch by terminating CAR T‐cell activity through small‐molecule‐induced apoptosis. Small‐molecule switch technologies for CAR T cells provide the advantage of controllable activity and enhanced safety. However, they may also entail risks, including CRS, neurotoxicity, drug‐related toxicity, variable efficacy, and potential drug interactions. Of particular concern is the irreversible activation of certain apoptosis‐inducing switches once triggered.

Future work on small‐molecule switches for CAR T cells must focus on a few things. Further designs should have lower immunogenicity and be chemically stable in biological systems. To achieve clinical application, more precise preclinical data on activation/inactivation concentrations and drug safety are required. Additionally, optimizing the pharmacokinetic properties of both switch molecules and engineered CAR T cells is essential for achieving predictable and reliable control in patients.

As these technologies mature, small‐molecule switches will broaden the therapeutic window of CAR T‐cell therapy, thus facilitating use in wider patient populations and diseases. The combination of precision chemical control and cellular immunotherapy is a significant transformation in cancer treatment. This provides an unprecedented opportunity to achieve a balance between potent antitumour activity and controllable safety profiles.

**Figure 2:**
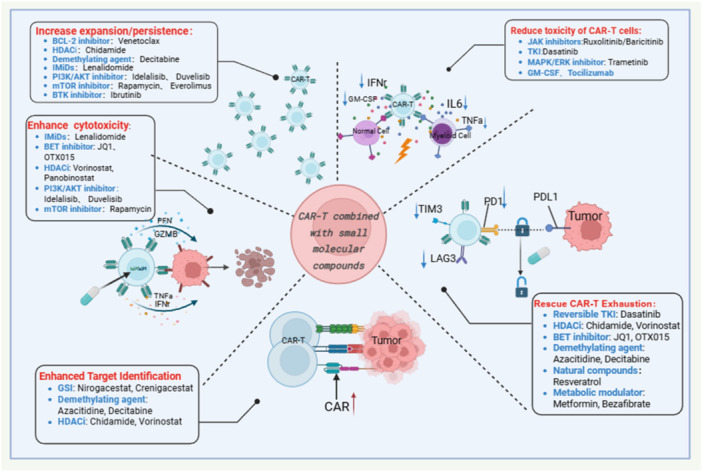
CAR T cells combined with small‐molecule compounds. Created in BioRender. rangzi, Y. (2026), https://BioRender.com/wsui07q.

To illustrate the utility of these strategies in aiding clinical decision‐making, small molecule compounds are classified into several major functional themes (Figure 2). First, enhancing the in vivo persistence and expansion of CAR T cells is a key determinant of durable remission and relapse prevention. Drugs that promote long‐term maintenance of memory‐like phenotypes and functional fitness include BCL‐2 inhibitors (e.g., venetoclax); epigenetic modulators, such as HDAC inhibitors (e.g., chidamide, vorinostat) and DNA demethylating agents (e.g., decitabine, azacitidine); immunomodulatory drugs (IMiDs), such as lenalidomide; and signalling pathway inhibitors, including BTK inhibitors and PI3K/AKT/mTOR inhibitors. Second, enhancing the direct cytotoxic activity of CAR T cells is a key strategy to improve antitumour efficacy. Related small molecules enhance the killing ability of CAR T cells through mechanisms that regulate the release of effector molecules, cell metabolism, or transcriptional programmes; representative drugs include immunomodulatory drugs (IMiDs), bromodomain and extraterminal domain (BET) inhibitors (such as JQ1, OTX015), histone deacetylase (HDAC) inhibitors, and PI3K/AKT/mTOR pathway inhibitors. Third, reversing CAR T‐cell exhaustion and restoring functional activity is key to facilitating durable efficacy. Approaches include the use of reversible TKI inhibitors (e.g., dasatinib), epigenetic modulators (HDAC and BET inhibitors), DNA demethylating agents, and metabolic regulators (e.g., metformin, bezafibrate) to correct exhaustion‐associated signalling and metabolic dysregulation. Fourth, the preventive approach primarily aims to enhance treatment tolerance by mitigating the severity of treatment‐related toxicity. JAK inhibitors (e.g., ruxolitinib) and GM‐CSF—neutralizing antibodies to suppress cytokine‐driven hyperinflammation, MAPK/ERK inhibitors, and TKIs (e.g., dasatinib) that act as “pharmacological switches” to transiently attenuate CAR T‐cell activity, enabling precise toxicity control. Finally, improving target recognition ability and modulating the TME are crucial for overcoming immunosuppressive barriers. GS inhibitors (e.g., nirogacestat, crenigacestat) enhance CAR T‐cell targeting and antitumour activity by increasing the surface availability of target antigens on tumour cells. And epigenetic modulators and metabolic regulators are utilized to reshape hostile immune microenvironments and synergistically enhance the efficacy of CAR T cells.

In conclusion, both preclinical and clinical research have explored combination therapy regimens. By using the programmable and controllable nature of small‐molecule drugs, these strategies aim to increase CAR T‐cell therapy efficacy precisely, mitigate associated toxicity, and overcome mechanisms of drug resistance. The ultimate objective is to achieve a synergistic therapeutic outcome, often referred to in terms of “1 + 1 > 2” (Figure 2). Consequently, the use of small‐molecule compounds combined with CAR T cells is adding versatility and potency to T cells beyond a simple additive effect towards an intelligent, controllable, and synergistic paradigm. This evolving approach is expected to overcome the current limitations of CAR T‐cell therapy and may bring new hope for a broader group of cancer patients.

## 3 Discussion

The integration of small‐molecule drugs with CAR T‐cell therapy enables a transition from single‐agent monotherapy to a multimodal precision intervention (Table 1). This strategic combination aims to precisely regulate the activity of CAR T cells and the TME through pharmacological means, in order to overcome the main limitations of cell immunotherapy. Compared with permanent genetic modifications, small‐molecule drugs provide unique plasticity and controllability to CAR T‐cell therapy, offering clear benefits in terms of reversibility, titratability, and spatiotemporal control. The pharmacological arsenal ranges from epigenetic modulators to metabolic reprogramming agents, signalling pathway inhibitors, and transcription factor modulators, providing multilayered solutions ready for core challenges, including CAR T‐cell exhaustion, an immunosuppressive TME, and antigen escape.

**Table 1:** Clinical trials of small‐molecule compounds combined with CAR T‐cell therapy.

Class	Compound	Target	Disease	Phase	NCT identifier	Efficacy	References
BCL‐2 inhibitor	Venetoclax	CD19	Ph^−^B‐ALL	Not Applicable	NCT06481241	/	
	Venetoclax + olverembatinib	CD19	Ph^+^‐ALL	Not Applicable	NCT06481228	/	
	Venetoclax + azacitidine	CD19/CD22	Ph^−^B‐ALL	II	NCT06078306	Post‐CD19/CD22 CAR T: CR 91.7%, MRD‐83.3% CRS: grade 1–2 (58.3%), grade 3 (1 case); no ICANS/neurotoxicity Median follow‐up: 17 months (range 10–22) 1‐year OS 81.8% (95% CI: 44.7–95.1) 1‐year LFS 81.8% (95% CI: 44.7–95.1) 1‐year CIR 18.2% (95% CI: 4.9–55.3).	[193]
	Venetoclax + ibrutinib	CD19	CLL	Not Applicable	NCT04640909	/	
Protein kinase inhibitor(TKI/BTK)	Ibrutinib	CD19	MCL	II	NCT06482684	/	
	Ibrutinib	CD19	CLL/SLL	I/II	NCT03960840	/	
	Ibrutinib	CD19	R/R B‐NHL	II	NCT05744037	/	
	Ibrutinib	CD19	R/R ALL、CLL、NHL	I/II	NCT01865617	4‐weeks ORR:83%; MRD^‐^ marrow response: 72% (flow)/61% (IGH) 1‐year OS 86%, PFS 59% CRS: low severity, mild toxicity MRD^−^ patients 1‐year PFS 100% vs. 59% (nonibrutinib)	[67]
	Ibrutinib	CD19	MCL	II	NCT04234061	4 months CR 80%；MRD^−^: 70% (flow), 40% (molecular) 12‐month PFS 75%, OS 100% CRS 75%: grade 1–2 (55%), grade 3 (20%) Neurotoxicity: reversible grade 1–2 in 10%.	[194]
	Ibrutinib	CD19	R/R BCL	III	NCT05020392	CRS: grade 1–2 (43.2%), grade 3 (2.7%)； Neurotoxicity: grade 3 (1 case)； ORR: 84.6% (with BTKi) vs. 66.7 (without BTKi)； CR: 61.5% (with BTKi) vs. 25% (without BTKi)	[195]
	Acalabrutinib	CD19	R/R MCL	II	NCT04484012	ORR 88%: CR 75% (*n* = 8), PR 13%(*n* = 1); 1‐year PFS 70% (95% CI: 22%–92%), OS 100% CRS:63% (grades 1–2) No ICANS.	[196]
	Acalabrutinib	CD19	R/R BCL	I/II	NCT04257578	After cell infusion: any grade CRS: 93%, ICANS: 57%, Day 30 post axi‐cel infusion:ORR:93% CR:71%; At a median follow‐up of 13.8 (range 1.7–29.1) months: 10 (73%) patients are alive and 9 are progression‐free.	[197]
	Acalabrutinib + rituximab	CD19	MCL	Ⅰ	NCT05495464	After one cycle of AR: PR:95% SD:5%; At day 30 post‐CAR‐T:ORR:100%, CR:95%, PR:5%; During AR: grade 3AEs (1 case), no grade 4 AEs; After brexu‐cel infusion: CRS: 5% (grade 3), 10% (grade 4); ICANS: 75% (grade 3 in 30% and grade 4 in 15%); all ICANS events resolved.	[198]
	Zanubrutinib	CAR T	R/R NHL	II	NCT05202782	/	
	Zanubrutinib	CD19	RS	II	NCT05873712	/	
	Zanubrutinib	CAR T	R/R B‐NHL	II	NCT06646666	/	
	Zanubrutinib + tislelizumab	CD19	R/R B‐NHL	II	NCT06695013	/	
	Zanubrutinib + obinutuzumab + lenalidomide	CD19/CD22	R/R B‐NHL	Not Applicable	NCT05797948	/	
	Dasatinib	CD19/BCMA	R/R B‐ALL/B‐NHL/MM	I	NCT04603872	/	
	Dasatinib	CD19/CD22	Ph+ ALL	I	NCT05523661	/	
	Dasatinib	CD19	R/R B‐ALL	I	NCT05993949	/	
HDACi	Chidamide	CD19	R/R B‐NHL	I/II	NCT05370547	/	
	Chidamide	CAR T	HIV‐1	Ⅰ	NCT03980691	/	
Demethylating agent	Decitabine	CD19/CD20	R/R B‐NHL	I/II	NCT04697940	/	
	Decitabine	CD19	R/R BCL	Ⅰ	NCT04850560	/	
	Decitabine + chidamide	CD19/CD20	R/R B‐NHL	I/II	NCT04553393	/	
Immunomodulator	Lenalidomide	BCMA	MM	II	NCT05032820	6 months CR ~ 63% (*n* = 38)； MRD^‐^87% at 6 months; 95% achieved MRD^−^ at some point Median follow‐up 10.7 months (IQR 8.3–12.3): Only 1 patient with disease progression; CRS: 81.6% (grade 1: 68.4%, grade 2: 13.2%); No ICANS.	[199]
	Lenalidomide	BCMA	MM	Ⅰ	NCT03070327	/	
	Lenalidomide	BCMA	MM	II	NCT03601078	Among the 8 pts who received Lenalidomide maintenance:CRR:75% ORR:100%	[200]
	Lenalidomide	BCMA	MM	III	NCT06045806	/	
	Lenalidomide	CD19	R/R CLL	I/II	NCT06762431	/	
	BiRd regimen (clarithromycin, lenalidomide and dexamethasone)	BCMA	MM	III	NCT04287660	BiRd induction (2 cycles): ORR 100% → PR 25% (5/20), VGPR 65% (13/20), CR 10% (2/20); Post‐CAR T: sCR 85%, VGPR 15%, MRD^−^ 80% (16/20); Follow‐up: median 25 months (range 5.8–80.1); 1 relapse (post‐ASCT 18 months); 2 deaths from infection (1 severe pneumonia at 51 months post‐ASCT, 1 COVID‐19 at 33 months post‐ASCT); 3‐year OS/PFS 100%; 5‐year OS 57.1% ± 24.9, PFS 55.6% ± 24.9; relapse: 3 and 5 years CIR 9.1% ± 8.7; median OS, PFS, DOR: not reached; CRS 95% (all grade 1–2); no ≥ grade 3 CRS; no ICANS.	[201]
	VRd regimen (bortezomib, lenalidomide and dexamethasone)	BCMA	pPCL	II	NCT05870917	/	
	VRd regimen (bortezomib, lenalidomide and dexamethasone)	BCMA	primary plasma cell leukaemia	II	NCT05979363	/	
	VRd regimen (bortezomib, lenalidomide and dexamethasone)	BCMA	MM	II	NCT05850286	Of the 8 evaluable patients:CRS:75% (25% grade 2 and 50% grade 1) and fully recovered; No ICANS; Median follow‐up after first CAR T infusion:CR + MRD^−^(*n* = 5), 1 VGPR, and 1 PR; 3 patients receiving second CAR T achieved CR + MRD^−^ with a median duration of MRD^−^ of 328 days.	[202]
	VRd regimen (bortezomib, lenalidomide and dexamethasone)	BCMA	MM	III	NCT04923893	/	
	VRd regimen (bortezomib, lenalidomide and dexamethasone)	BCMA	MM	II	NCT05860036	All treated patients achieved CR; In 14 patients with adequate follow‐up: all sustained MRD^−^ within 6 months; 3 pts sustained MRD^−^ up to 12 months.	[203]
	DVRd (daratumumab, bortezomib, lenalidomide and dexamethasone)	BCMA	MM	III	NCT05257083	/	
	DVRd (daratumumab, bortezomib, lenalidomide and dexamethasone)	BCMA	MM	II	NCT04133636	/	
	Epcor‐R2 (epcoritamab, lenalidomide and rituximab)	CD19	R/R B‐NHL	II	NCT06414148	/	
PI3K/AKT inhibitors	Duvelisib	CD19	R/R DLBCL	Ⅰ	NCT04890236	/	
	Duvelisib	CAR T	ALL/NHL	Ⅰ	NCT05044039	Day 30: ORR 71% (12/17), CR 47%; Day 100: ORR 64% (9/14), CR 50%; Best response: CR 71%, SD 18%, no PD; median onset day 5 (range 2–9):CRS: 76% (mostly grade 1, 65%), no grade 3–4; median of 7 days (range 4–10):ICANS: 41% (12% grade 3–4), median duration 5.5 days; median follow‐up 93 days (range 28–406); 53% in remission; median time to progression of 89 days (range: 28–182): PD: 35% (6/17)	[204]
mTOR inhibitors	Rapamycin	CD19	R/R LBCL/CLL	Ⅰ	NCT06528301	/	
	Rapamycin	CD33	AML	Ⅰ	NCT05105152	/	
JAK/STAT inhibitors	Itacitinib	CD19	DLBCL	II	NCT05757219	/	
	Itacitinib	CD19	R/R LBCL/FL	II	NCT04071366	Day 14, grade ≥ 2 CRS:17.4% (200 mg itacitinib twice daily) vs. 56.5% (placebo); Day 28, grade ≥ 2 ICANS: 8.7% (200 mg itacitinib twice daily) vs. 21.7% (placebo); IEC therapy efficacy: ORR at 6 months: 39.1% (200 mg itacitinib twice daily) vs. 26.1% (placebo)	[125]
	Ruxolitinib	CD123	R/R AML	Ⅰ	NCT06768476		
natural compounds	Quercetin + dasatinib	BCMA	R/R MM	II	NCT06940297	/	
γ‐Secretase inhibitors	LY3039478 (JSMD194)	BCMA	MM	Ⅰ	NCT03502577	Median follow‐up 36 months (95% CI: 26–NR); Most common ≥ grade 3 nonhematologic AEs: hypophosphatemia 78% (14/18), fatigue 61% (11/18), hypocalcemia 50% (9/18), hypertension 39% (7/18); 2 deaths reported outside 28‐day AE window; treatment‐related; dose up to 450 × 10^6^ CAR T cells; recommended phase 2 dose not reached.	[205]
	BMS‐986405 (JSMD194)	BCMA	MM	I/II	NCT04855136	/	

Nevertheless, the clinical translation of combination strategies faces multiple challenges. Complex drug interactions present a primary concern, as small‐molecule drugs may directly interfere with CAR T‐cell activation, proliferation, and effector functions. Toxicity overlaps risks complicating clinical implementation of combination therapies, whereby the toxicity of CAR T‐cell therapy may be compounded with the side effects of the small molecule. Defining the therapeutic window for combination therapy will be a question of dose exploration and the optimal timing of the drug combination. Finally, the immaturity of reversible control technologies may remain a barrier. Although small‐molecule switches have broad application prospects, they still require further validation regarding long‐term safety, immunogenicity, and reliability in human applications.

Future approaches should focus on mechanism‐driven precision combinations on the basis of a better understanding of tumour resistance and T‐cell dysfunction mechanisms. The development of “immunology‐specialized” small molecules using high‐throughput screening and AI‐assisted design is a promising way to minimize unintended negative effects on CAR T‐cell activity. Creative clinical trial designs using adaptive platforms could efficiently compare different combinations while achieving accurate patient stratification on the basis of biomarkers. Additionally, bringing next‐generation small‐molecule switches to clinical translation would achieve real‐time, reversible control of CAR T‐cell activity, transforming toxicity management from a crisis response to proactive prevention.

In conclusion, the combination of small‐molecule drugs with CAR T cells marks the entry of cancer treatment into an era of “engineerable” therapeutics. Future success will depend on close interdisciplinary collaboration to systematically resolve the complexities of combination therapy, ultimately benefiting a broader patient population through more intelligent, safer, and more effective anticancer regimens.

## 4 Conclusions

In this review, we summarized the key advances in the synergistic integration of small‐molecule drugs with CAR T‐cell therapy. This combination approach turns the cell immunotherapy from a single treatment into a precise, multi‐target therapy, making it more effective, longer persistence and safety. As we learn more about the molecular mechanisms of immunotherapy and the TME, more precise and personalized combination treatments will become a key part of future cancer therapy, helping more patients receive effective care.

## Author Contributions

**Rangzi Yi:** writing – review and editing. **Zijian Zhang:** writing – review and editing. **Yang Yang:** conceptualization, supervision. **Haichuan Zhu:** conceptualization, writing – review and editing, supervision.

## Ethics Statement

The authors have nothing to report.

## Consent

The authors have nothing to report.

## Conflicts of Interest

The authors declare no conflicts of interest.

## Data Availability

The authors have nothing to report.
